# Point-of-Care Ultrasound Diagnosis of a Crohn’s Disease-Related Intraabdominal Abscess in the Emergency Department

**DOI:** 10.7759/cureus.14290

**Published:** 2021-04-04

**Authors:** Kevin Rivera, Gabriel Cabrera, Eric J Kalivoda

**Affiliations:** 1 Emergency Medicine, Hospital Corporation of America (HCA) Healthcare/University of South Florida Morsani College of Medicine Graduate Medical Education (GME) Consortium, Brandon Regional Hospital, Brandon, USA

**Keywords:** point-of-care ultrasound, ultrasonography, intraabdominal abscess, crohn's disease, inflammatory bowel disease, emergency department

## Abstract

The emergency department (ED) diagnosis of Crohn’s disease (CD)-associated complications is typically established with abdominopelvic computed tomography imaging. Ultrasonography has been suggested as an effective alternative modality for diagnosing several CD complications, including intraabdominal abscesses. We report the identification of a CD-related intraabdominal abscess by emergency physician (EP)-performed point-of-care ultrasound (POCUS). This case highlights the feasibility of EPs integrating POCUS into the clinical decision-making for patients with inflammatory bowel disease in the ED.

## Introduction

Crohn’s disease (CD) is an inflammatory bowel disease (IBD) subtype which is characterized by chronic transmural inflammation of the gastrointestinal tract with severe complications of intestinal fistulas, strictures, obstruction, perforation, and intraabdominal and pelvic abscesses [1]. IBD-related emergency department (ED) visits have continued to rise, therefore, emergency physicians (EPs) should remain aware of these life-threatening complications [2,3]. EPs often rely on computed tomography (CT) as a first-line imaging modality for ED evaluation, however, the cumulative lifetime radiation exposure for CD patients is highly concerning due to CT overutilization [4]. Ultrasonography (US) is an alternative non-invasive modality for the diagnosis of a multitude of CD complications (including fistulas, strictures, and intraabdominal abscesses), while maintaining indispensable advantages of widespread availability, lower cost, and avoidance of radiation exposure [5-9]. The incidence of intraabdominal and pelvic abscesses in CD is approximately 10-30% over the course of a patient’s illness [1,8]. US has been previously demonstrated as an accurate diagnostic tool for CD-related intraabdominal abscesses [5-8]. To the best of our knowledge, this is the first case report to describe EP-performed point-of-care ultrasound (POCUS) identification of a CD-related intraabdominal abscess.

## Case presentation

A 28-year-old male with a past medical history of nephrolithiasis, cholecystectomy and Crohn’s disease (CD) presented to the ED with one week of gradual-onset, constant, and mild-severity bilateral lower abdominal pain with associated dysuria. He denied fevers, chills, nausea, vomiting, diarrhea, constipation, dark or bloody stools, flank pain, back pain, testicular pain or swelling, genital lesions or discharge, hematuria, urinary frequency and urgency, or any other associated symptoms. Notably, the patient also reported a history of CD treatment failure with multiple immunomodulator and biologic medications, however, he had no relevant surgical history as a consequence of treatment failures. Upon arrival, the patient was afebrile, blood pressure of 134/75, heart rate 96 beats per minute, respiratory rate 18 breaths per minute, and oxygen saturation of 100% on room air. On physical examination, he was in no acute distress, well-appearing, and mild tenderness to palpation was present in bilateral lower abdominal quadrants without rebound, guarding, or peritoneal signs. Notably, there was no flank or costovertebral angle tenderness appreciated. The remainder of the physical examination was unremarkable.

**Figure 1:**
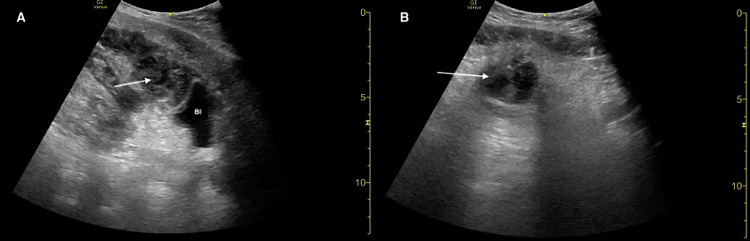
Point-of-care ultrasound demonstrating a Crohn’s disease-related intra-abdominal abscess (arrows) in sagittal (A) and transverse (B) planes. Bl: bladder.

The patient’s clinical presentation was most concerning for a gastrointestinal complication related to Crohn’s disease versus a genitourinary process. Laboratory studies, urinalysis, pain medications, and intravenous (IV) fluids were ordered. An ultrasound fellowship-trained EP then performed a focused abdominal POCUS examination. Given that the patient had a known history of nephrolithiasis, renal POCUS was performed and unremarkable for any findings of urinary obstruction (i.e., hydronephrosis or kidney stones). Incidentally, during assessment of the urinary bladder, POCUS revealed the presence of a round hypoechoic lesion with internal echoes and posterior acoustic enhancement located superior to the urinary bladder, highly suggestive of an intraabdominal abscess (Figure 1).

**Figure 2:**
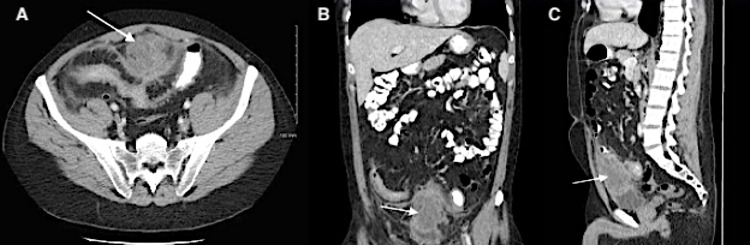
Computed tomography of abdomen/pelvis demonstrating an intraabdominal abscess (arrows) secondary to complication of Crohn’s disease in axial (A), coronal (B), and sagittal (C) planes.

Broad-spectrum IV antibiotics to cover for an intra-abdominal infection were ordered based upon the abnormal POCUS findings. The case was subsequently discussed with gastroenterology who requested obtaining emergent abdominopelvic computed tomography (APCT) imaging. APCT confirmed a complex fluid collection (measuring 5.2 x 5.2 x 6.8 cm) in the anterior pelvis, just superior to the bladder dome and adjacent to an inflamed loop of distal ileum, most concerning for an abscess with surrounding inflammatory stranding (Figure 2). Urinalysis was positive for nitrites however with urine white blood cells 0-5 (0-5/high-power field). Laboratory analysis was unremarkable for leukocytosis or other acute clinically significant abnormalities.

The patient was admitted for additional IV antibiotics and further management by gastroenterology (GI) and interventional radiology (IR). IR ultimately performed CT-guided percutaneous drainage of the intraabdominal abscess, yielding approximately 35 milliliters of purulent fluid. His hospital course was uncomplicated. The patient was discharged home on hospital day 3 with plan for an oral antibiotic regimen of Ciprofloxacin and Metronidazole, daily Prednisone, and instructions for outpatient GI follow-up.

## Discussion

The ED evaluation of symptomatic CD patients frequently necessitates an APCT to identify clinically relevant complications such as fistulas, strictures, obstruction, perforation, and intraabdominal abscesses [1,4]. APCT imaging is typically warranted given the high rates of detecting serious CD complications ranging from 29-47% and the identification of intraabdominal abscesses in 9-18% of these ED encounters [4,10-12]. There is an overall trend towards increased CT usage with concern for lifetime radiation exposure in this patient population [4]. US has been proposed as an alternative imaging modality for the assessment of CD disease activity, severity, and complications [9]. Based on the results of three previous studies, the detection of CD-related intraabdominal abscesses with US has pooled sensitivity and specificity of 84% and 93%, respectively [5-7,9]. Contrast-enhanced US has reported sensitivity and specificity of 97% and 100%, respectively, for the diagnosis of CD-related intraabdominal abscesses, while also importantly demonstrating accuracy in the differentiation between inflammatory phlegmons [13]. Nevertheless, there are notable limitations of US that may preclude its routine implementation for IBD patients presenting to the ED. Previous studies have demonstrated a higher sensitivity of US for superficial intraabdominal abscesses versus deep pelvic abscesses and other anatomic locations difficult to visualize due to bowel gas; additionally, smaller intraabdominal abscesses are likely to be more challenging to recognize with US [8,9]. The role of EP-performed POCUS in the ED diagnosis of CD complications is yet to be established, however, will likely evolve as we better understand the implications of disproportionate CT utilization in IBD patients. The clinical management of CD is a multidisciplinary effort between specialists in emergency medicine, gastroenterology, general surgery, and radiology, therefore, the development of best practice guidelines regarding ED imaging for this patient cohort would be instrumental in improving patient care [4].

Multiple studies have investigated whether clinical variables can provide predictive information regarding the likelihood of significant APCT findings [11,12,14,15]. The presence of abnormal vital signs (heart rate > 100 beats/minute and fever > 37.5°C) and/or abnormal laboratory markers (leukocyte count > 12,000/mm^3^, neutrophil-to-lymphocyte ratio > 11.75, and C-reactive protein > 2.5 mg/dL) has been reported as independent positive predictors of CD complications on APCT [11,12,14,15]. Current corticosteroid and/or biologic use has been identified as an independent negative predictor of CD complications, particularly intraabdominal abscess formation [11,15]. Clinical decision-making tools integrating these variables have been developed to assist the EP in risk stratifying those patients at highest risk of CD complications [14,15]. Interestingly, based on existing diagnostic scoring systems, our patient might have been classified into a low-risk group for CD complications because of normal clinical parameters, but the abnormal POCUS finding of an intraabdominal abscess prompted an adjustment in risk stratification to warrant APCT imaging. This case highlights the potential utility of incorporating EP-performed POCUS into the clinical decision-making in the evaluation of IBD patients.

## Conclusions

To the best of our knowledge, this is the first case report to describe the identification of a CD-related intraabdominal abscess with EP-performed POCUS. POCUS is a promising non-invasive diagnostic tool for EPs in the rapid detection of CD complications such as intraabdominal abscesses, with the critical advantage of avoiding ionizing radiation exposure, although with significant limitations that should be acknowledged. This report illustrates the feasibility for EPs to incorporate POCUS into risk stratification algorithms to determine the likelihood of abnormal APCT imaging. Further prospective ED-based studies are needed to establish the clinical utility and effectiveness of EP-performed POCUS in detecting life-threatening complications of CD.
